# Improving radiologist's ability in identifying particular abnormal lesions on mammograms through training test set with immediate feedback

**DOI:** 10.1038/s41598-021-89214-3

**Published:** 2021-05-10

**Authors:** Phuong Dung (Yun) Trieu, Sarah J. Lewis, Tong Li, Karen Ho, Dennis J. Wong, Oanh T. M. Tran, Louise Puslednik, Deborah Black, Patrick C. Brennan

**Affiliations:** 1 Faculty of Medicine and Health, The University of Sydney, Level 7 - D18, Susan Wakil Health Building, Sydney, NSW 2006 Australia; 2 Vietnam Health Strategy and Policy Institute, Department of Health, 196 Ho Tung Mau Street, Cau Giay District, Hanoi, Vietnam; 3 University of Medicine and Pharmacy, 215 Hong Bang, District 5, Ho Chi Minh City, Vietnam; 4 St Matthew′s Catholic School, Mudgee, NSW Australia

**Keywords:** Breast cancer, Diagnosis

## Abstract

It has been shown that there are differences in diagnostic accuracy of cancer detection on mammograms, from below 50% in developing countries to over 80% in developed world. One previous study reported that radiologists from a population in Asia displayed a low mammographic cancer detection of 48% compared with over 80% in developed countries, and more importantly, that most lesions missed by these radiologists were spiculated masses or stellate lesions. The aim of this study was to explore the performance of radiologists after undertaking a training test set which had been designed to improve the capability in detecting a specific type of cancers on mammograms. Twenty-five radiologists read two sets of 60 mammograms in a standardized mammogram reading room. The first test set focused on stellate or spiculated masses. When radiologists completed the first set, the system displayed immediate feedback to the readers comparing their performances in each case with the truth of cancer cases and cancer types so that the readers could identify individual-based errors. Later radiologists were asked to read the second set of mammograms which contained different types of cancers including stellate/spiculated masses, asymmetric density, calcification, discrete mass and architectural distortion. Case sensitivity, lesion sensitivity, specificity, receiver operating characteristics (ROC) and Jackknife alternative free-response receiver operating characteristics (JAFROC) were calculated for each participant and their diagnostic accuracy was compared between two sessions. Results showed significant improvement among radiologists in case sensitivity (+ 11.4%; *P* < 0.05), lesion sensitivity (+ 18.7%; *P* < 0.01) and JAFROC (+ 11%; *P* < 0.01) in the second set compared with the first set. The increase in diagnostic accuracy was also recorded in the detection of stellate/spiculated mass (+ 20.6%; *P* < 0.05). This indicated that the performance of radiologists in detecting malignant lesions on mammograms can be improved if an appropriate training intervention is applied after the readers’ weakness and strength are identified.

## Introduction

With over 2 million new cases of breast cancer diagnosed worldwide in 2018, breast cancer has become the most commonly occurring cancer in women and the second most common cancer overall^1^. Patient treatment outcomes rely heavily on accurate interpretation of radiologists for the early detection of abnormal lesions on mammography, which is currently the most popular X-ray imaging method used for both diagnostic and screening purposes of breast cancer. It has been shown that the diagnostic accuracy of radiologists in screening mammograms varied significantly across countries ranging from below 50% in developing countries to over 80% in developed nations^2–4^. Although reasons could partly stem from differences in patient populations, variability has also been documented in the interpretative skills of radiologists. Whilst many studies have been conducted to find explanations of this variability, such as mammogram reading volume, malpractice anxieties, and other radiologist characteristics^5–7^, only a few studies have concentrated on practical approaches to improve diagnostic performances of readers. Furthermore, the educational components that contributed most to the observed effects are challenging to determine^8,9^.

As a result of the significant variability in diagnosis, mammography quality standards in many countries include mandatory continuing medical education units in mammogram reading annually for breastscreen readers^10^. This training practice could focus specifically on skills assessment using test sets with a mix of normal and abnormal findings and feedback options. Assessment methods could compare the performance of radiologists with experts to provide them with feedback on their diagnostic skills and identify areas for improvement^11^.

The Breastscreen REader Assessment STrategy (BREAST) has been developed as the training and self-assessment tool for radiologists in Australia since 2011. It monitors the performance of readers in detecting abnormalities on mammograms using a novel, web-based software that offers immediate feedback to radiologists and identifies all individual-based errors^11^. Over the last eight years, BREAST has engaged more than 80% of breastscreen clinicians (radiologists, breast physicians, registrars) across all states in Australia and been evaluated as one of the best professional development tools for radiologists^11^. It has also been shown that the regular completion of the BREAST test sets improved radiologists’ test based performances^12,13^. In recent years, BREAST has been introduced to Vietnam, a developing country in South East Asia, through workshops for radiologists, radiology residents and data showed that Vietnamese radiologists displayed a low mammographic cancer detection of 48% compared with 81% for their counterparts in Australia and Singapore^4^. Importantly, it is found that the detection rate of Vietnamese radiologists for specific types of lesions such as spiculated masses or stellate lesions on mammograms were lower than calcification, discrete mass or asymmetric density^14^.

The aim of this study is to explore the improvement in diagnostic accuracy of Vietnamese radiologists in detecting specific types of cancers on mammograms after undertaking a training test set designed to improve screening mammography interpretation. We tested the hypotheses that the training sets with individualised feedback would increase radiologists’ ability to identify particular cancerous mammographic findings requiring further assessment.

## Results

**Figure 1:**
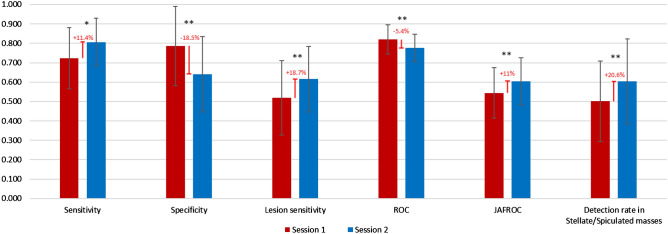
Comparison of radiologists’ performances between two reading sessions (Session 1 with training set and Session 2 with original BREAST test set).

Radiologists obtained significantly higher scores in sensitivity (+ 11.4%; *P* < 0.05), lesion sensitivity (+ 18.7%; *P* < 0.01), JAFROC (+ 11%; *P* < 0.01) in the second set compared with their performances with the first training set. The improvement in diagnostic accuracy among readers was also recorded in the detection of stellate/spiculated mass (+ 20.6%; *P* < 0.05) after the first training set. There were increases in the recall rate (+ 27%; *P* = 0.004) and the false positive (false recall) rate (+ 15%; *P* = 0.001) in the second set compared with the first set. The specificity and ROC, however, decreased in the second session (− 18.5%; *P* < 0.01) and ROC (− 5.4%; *P* < 0.01) compared with the first session. A moderate reliability among radiologists was found across performance results in the first set (ICC = 0.628 with 95% confident interval = 0.335–0.817) and second test set (ICC = 0.566 with 95% confident interval = 0.223–0.786). No statistical difference demonstrated in the reading time of participants between two reading sessions (6628 s vs. 6104 s; *P* > 0.05), indicating that radiologists did not spend more time making decisions, or searching for cancers, in the second test set (Fig. 1).

Male readers showed substantial decrease in reading time in the second set compared with the time they spent in the first set (− 18.2%; *P* = 0.03). Both male and female readers were recorded an improvement in identifying stellate/spiculated masses (+ 28.5%; *P* = 0.046 and + 17.5%; *P* = 0.04). Improved performances in these types of lesions after the first set were also found among readers with less than 2 years of reading mammograms (+ 37.8%; *P* = 0.02), reading ≥ 20 mammograms per week (+ 20.3%; *P* = 0.02), learning mammogram interpretation from senior colleagues or attending training courses (+ 15.3%; *P* = 0.02) (Table 1).

**Table 1:** Comparisons of the performances of readers in different characteristics in two reading sessions (Session 1: Training set, Session 2: Original test set).

Category (number of readers)	Mode	Reading time (s)	Detection rate in stellate/spiculated masses
Male (6)	Session 1	7300	0.422
	Session 2	5974	0.542
	**Difference (%)**	**−** **18.2**	**28.5**
	*P* value	0.028	0.046
Female (19)	Session 1	6415	0.526
	Session 2	6145	0.618
	**Difference (%)**	**−** **4.2**	**17.5**
	*P* value	0.629	0.040
Years of reading mammograms < 2 (14)	Session 1	6369	0.382
	Session 2	6221	0.527
	**Difference (%)**	**−** **2.3**	**37.8**
	*P* value	0.594	0.017
Years of reading mammograms ≥ 2 (11)	Session 1	6956	0.653
	Session 2	5955	0.693
	**Difference (%)**	**−** **14.4**	**6.2**
	*P* value	0.286	0.182
Reading < 20 mammograms per week (14)	Session 1	6799	0.420
	Session 2	5891	0.500
	**Difference (%)**	**−** **13.4**	**19.0**
	*P* value	0.177	0.124
Reading ≥ 20 mammograms per week (11)	Session 1	6410	0.604
	Session 2	6375	0.727
	**Difference (%)**	**−** **0.5**	**20.3**
	*P* value	0.722	0.021
Learning from senior colleagues or attending training courses (7)	Session 1	7099	0.588
	Session 2	6848	0.679
	**Difference (%)**	**−** **3.5**	**15.3**
	*P* value	1	0.018
Self-learning (18)	Session 1	6444	0.467
	Session 2	5815	0.569
	**Difference (%)**	**−** **9.8**	**21.8**
	*P* value	0.248	0.061

## Discussion

In this study, we explored the effect of a training set designed to improve interpretive performance of radiologists in Vietnam in detecting specific types of cancers on screening mammograms. After undertaking a training set, the scores of radiologists in case sensitivity, lesion sensitivity and detection rate of stellate or spiculated masses were significantly improved.

Previous studies showed that the interpretive performance varies considerably among radiologists in different countries^2–4^. According to the literature, approximately 62% of radiologists interpret mammograms as part of their workload, but only 10.5% consider themselves breast imaging specialists^15^. In Vietnam, a developing country in SouthEast Asia with no population-based breast screening program, the number of breast imaging specialists is less than 5% and the diagnostic performances of Vietnamese radiologists in mammograms were found to be significantly lower that their counterpart in developed countries such as Australia or Singapore^4^. Whilst caution is to be exercised about generalisations surrounding international performance comparisons due to other confounding factors, there is strong evidence that greater support within the Vietnamese postgraduate medical education system for junior doctors in breast imaging is required. In developed countries, high emphasis is placed on medical graduates developing competencies after their general medical education, and professional colleges of radiology play a central role with this. However, currently in Vietnam there is no official training course for breast screen radiologists to learn and advance their knowledge in this field. In the absence of an authorized training regimen, the implementation of educational training programs such as BREAST to clinical practices could have important complementary roles.

Our report is the first published study to date to use a training set designed for a specific group of readers as an intervention to improve mammography interpretive performance of radiologists^16^. The findings of this study have established that after undertaking the training set designed to improve stellate/spiculated mass detection (which was the lesion type most commonly missed by)^14^, Vietnamese radiologists obtained significantly higher scores in case and lesion sensitivity. The improvement rates ranged from 6 to 30% with differences being possibly linked to a myriad of factors. Radiologists who read less than 20 cases per week showed no significant improvement in detecting of stellate or spiculated masses compared to radiologists with higher number of cases reading per week. This is in line with the findings from a previous study showing the positive relationship between the number of mammographic images read annually and reader performances^17^ suggesting that number of cases reading might play more important role in diagnostic performances of radiologists.

Other studies showed the important impact of training programs, even though the approach described in our study is different from those employed by others. Linver and colleagues in 2002^18^ reported that attendance at an extensive 3- to 4-day instructor-led educational program in breast imaging resulted in considerable improvement in sensitivity. This intensive training used a pre-intervention and post-intervention clinical audit to measure the change, with 12 radiologists participating from a single practice. Earlier in 1992, Berg et al. used a single test set with all cases containing cancer and the experiment showed an increase in sensitivity. Testing was administered immediately before and after the teaching intervention and again 2–3 months after the first intervention for a small subset of participants^9^.

Our study has a number of benefits not realised in previous study designs using education interventions. Unlike many previous studies using the film-screen mammograms or photographic slides, our training sets were digital mammograms collected from breastscreen services and the reading platform allowed radiologists to be able to manipulate the mammograms freely. Compared with previous studies which used interventional methods taking from 3 days to 3 months of radiologists’ time to witness the improvement, our training test sets took readers no more than 3 h and this had a benefit in saving readers’ time and decreasing readers’ fatigue. Moreover, immediate feedback was provided to readers through the BREAST system after they completed the training sets so that readers could compare their interpretations and experts’ detections with biopsy-proven truth and increase the practical knowledge. Feedback‐seeking behaviour is highlighted as an important learner characteristic as it sets the stage for a successful exchange of information during the educational encounter. Additionally, the learners’ emotional reaction to the feedback, including fear of receiving “negative” feedback or appearing incompetent, played a significant role in their likelihood of seeking feedback. Eva et al.^19^ demonstrated that learner confidence and fear of not appearing knowledgeable are important considerations for feedback receptivity. In our training system, radiologists received the feedback of their diagnostic accuracy individually and confidentially which could relieve the fears and increase the confidence of the readers.

Along with using training test sets in clinical practice, a great effort has been made in the development of computer-aided diagnosis (CAD) to assist radiologists better detect early breast cancer lesions on digital images. CAD is described as a computer-based tool that highlights suspicious regions on a mammogram and thus alerts a reader to a location in a mammogram where computerized analysis suggests that abnormalities may exist. The general consensus is that CAD may provide up to 20% of improvement in breast cancer detection rates^20^, however significant controversies regarding patient outcomes remain: A study of 323,973 women between 2003 and 2009 found no evidence of increased breast cancer detection rates with CAD as compared to those without CAD (mammography sensitivity was 85.3% with and 87.3% without CAD while specificity was 91.6% with and 91.4% without CAD) and concluded there was no benefit with CAD; Bargolla et al.^20^ stated that CAD did not detect any cancers that the radiologist did not initially perceive; Gross et al.^21^ suggested that the use of CAD or digital mammography had limited effectiveness for older, average-risk women and higher costs related with the adoption of such technologies without a corresponding better outcomes; Onega et al.^22^ mentioned that radiologists had overall more favourable perceptions of double reading by a colleague rather than single reading with CAD. Therefore, with ongoing CAD controversies and artificial intelligent being at an early stage of implementation, the usage of training sets through online platform will continue to play an important role to optimise radiologic performances within screening mammography.

Assessing the level of suspiciousness in a region is an important aspect of breast screening and results from this study included a specific focus on lesion location sensitivity is clinically relevant for tracking improvements in mammographic reading performance. When a woman is recalled for an abnormal finding, the additional imaging assessment (e.g. diagnostic magnification views, ultrasound) focuses on the region of interest (although the extent of disease is addressed during the diagnostic evaluation). Therefore, it is important to detect and identify the correct abnormal appearances and their locations. This work shows that after undertaking a specifically designed training set, individuals showed significant improvements in detecting stellate or spiculated masses on mammograms. This highlights the effectiveness of using specific mammogram training sets targeted for specific learners and certain types of cancer. Considering the lack of radiology workforce in Vietnam to facilitate face to face learning, we believe these results show that online training programs, particularly for young radiologists, are an effective training strategy. Breast screening programs in many nations including Australia maintain competency through regular test set readings such as that made available through the Breastscreen REader Assessment STrategy (BREAST).

It is important to recognize that sensitivity and specificity exist in a state of balance in real-life screening. Improved screening test sensitivity is often accompanied by reduced specificity, leading to increased false-positive outcomes or vice versa. Radiologists in this study showed a decrease in specificity in the second read when they read cancer-enriched datasets, which had different cancer prevalence to real-field data (30% in the study dataset versus < 1% in real-field data). Literature showed that inclusion of a large number of malignancies in the test set would introduce “context bias” that tends to increase sensitivity and decrease specificity^23^. The reduction in specificity was most likely linked to the increase in the recall rate (+ 27%) and the false positive (false recall rate) (+ 15%) in the second set compared with the first set. Furthermore, our study focused on improving the detection of specific type of abnormal findings and therefore did not take account on selecting appropriate normal cases for training to improve the false positive rates of radiologists. This is an interesting aspect to explore in the future.

It should also be acknowledged that there was a different composition of cancer types in the first set and second set since the first set was used with the aim of training radiologists in identifying stellate and spiculated masses. The radiologists were only aware of the truth of cancer locations after they completed reading the training set and the second set was a more typical test set consisting of a greater range of cancer appearances with 50% of cancers being stellate and spiculated lesions. In this preliminary study, the sample size was limited to a group of 25 Vietnamese radiologists, but even with this number, the results still showed significant improvement in the cancer detection rate suggesting that these results could be relevant to a larger group of radiologists with different backgrounds and experiences.

In conclusion, cancer detection rate of radiologists on mammograms can be improved by using appropriate training interventions based on actual clinical cases with findings that are frequently misinterpreted. This indicates that the training systems with digital mammograms can be a powerful tool for doctors to improve their diagnostic level in detecting specific types of cancer appearances once those weaknesses have been identified.

## Methods

This study was performed in accordance with relevant guidelines and regulations. The study received the Institutional Review Board approval (IRB number: 23/UMP-BOARD) from Ho Chi Minh city University of Medicine and Pharmacy for data collection in Vietnam and ethical approval from Human Research Ethics Committee of the University of Sydney (2019/013) for utilizing the BREAST databank. The need for patient consent for the use of anonymized mammograms was waived and the informed consent was obtained from radiologists who were participants in the study.

### Case selection

Two sets of mammograms were gathered from the BREAST database to prepare for this study. From the previous study^14^, our team identified that spiculated masses or stellate lesions were commonly missed by Vietnamese radiologists compared with other types of abnormal lesions on mammograms. Therefore, a mammogram training set including 38 normal and 22 biopsy-proven cancer cases containing 19 stellate/spiculated masses and 3 architectural distortion were built as the first reading set. The second set was an original BREAST test set of mixed cancer features and consisted of 40 normal and 20 cancer cases with 10 stellate/spiculated masses, 4 asymmetric density, 4 microcalcifications, 1 discrete mass and 1 architectural distortion. Cases in the second set were different from the ones used in the first set and had higher level of difficulty (Table 1) in order to be challenging to participants, and thus offer both assessment as well as training values. The rate of cancer cases with high breast density (level C and D) in the first set was 36% and 75% in the second set. The average size of lesions in first and second sets were at 11.2 mm and 11.7 mm respectively. The mammographic cases were selected by two senior radiologists with more than 25 years of experience working in Australian BreastScreen services. Normal cases were verified via a negative two-year follow up of the initial images whilst cancer cases were confirmed by the pathology reports. Each examination consisted of two-view mammograms of both breasts, a cranio-caudal (CC) and mediolateral oblique (MLO). Any cases with a post-biopsy marker or a surgical scar visible was not included. Details of cancer cases in set 1 and 2 are described in Table 2.

**Table 2:** Description of cancer cases used in the first session (training set) and the second session (an original BREAST test set).

Set	Case	Breast density*	Cancer type	Lesion size (mm)	Difficult value	Set	Case	Breast density*	Cancer type	Lesion size (mm)	Difficult value
1	1	C	Architectural distortion	26	0.18	2	1	C	Architectural distortion	20	0.39
1	2	C	Architectural distortion	11	0.04	2	2	C	Calcification	7	0.09
1	3	B	Architectural distortion	13	0.16	2	3	C	Calcification	9	0.25
1	4	B	Stellate/spiculated mass	10	0.11	2	4	C	Calcification	14	0.40
1	5	B	Stellate/spiculated mass	12	0.18	2	5	C	Calcification	15	0.32
1	6	C	Stellate/spiculated mass	12	0.07	2	6	C	Discrete mass	10	0.31
1	7	B	Stellate/spiculated mass	10	0.53	2	7	A	Asymmetric density (AD)	11	0.25
1	8	B	Stellate/spiculated mass	11	0.13	2	8	B	Asymmetric density (AD)	7	0.19
1	9	D	Stellate/spiculated mass	10	0.29	2	9	B	Asymmetric density (AD)	8	0.30
1	10	D	Stellate/spiculated mass	9	0.07	2	10	C	Asymmetric density (AD)	15	0.19
1	11	B	Stellate/spiculated mass	20	0.16	2	11	C	Stellate/spiculated mass	18	0.07
1	12	B	Stellate/spiculated mass	8	0.27	2	12	B	Stellate/spiculated mass	14	0.22
1	13	B	Stellate/spiculated mass	9	0.40	2	13	C	Stellate/spiculated mass	7	0.07
1	14	C	Stellate/spiculated mass	10	0.11	2	14	B	Stellate/spiculated mass	10	0.36
1	15	B	Stellate/spiculated mass	8	0.87	2	15	C	Stellate/spiculated mass	7	0.35
1	16	B	Stellate/spiculated mass	7	0.22	2	16	C	Stellate/spiculated mass	11	0.92
1	17	B	Stellate/spiculated mass	9	0.20	2	17	C	Stellate/spiculated mass	18	0.72
1	18	B	Stellate/spiculated mass	10	0.22	2	18	C	Stellate/spiculated mass	7	0.13
1	19	B	Stellate/spiculated mass with AD	12	0.36	2	19	C	Stellate/spiculated mass	15	0.15
1	20	B	Stellate/spiculated mass with AD	9	0.47	2	20	C	Stellate/spiculated mass with AD	10	0.34
1	21	C	Stellate/spiculated mass with AD	14	0.24						
1	22	C	Stellate/spiculated mass with AD	7	0.18						
Mean (all cancer types)				11.2	0.25	Mean (all cancer types)				11.7	0.30
Mean (stellate/spiculated mass ± AD)				10.4	0.27	Mean (stellate/spiculated mass ± AD)				11.7	0.33

Difficult values were calculated from the BREAST database which were based on the rate of 147 Australian radiologists (45 in set 1 and 102 in set 2) failing to detect cancer locations on the mammograms (the higher values represent the more difficult cases).

*A: fibrograndular tissue < 25%, B: fibroglandular tissue 25–50%, C: fibroglandular tissue 51–75%, D: fibroglandular tissue > 75%.

### Participants

Twenty-five doctors (21 radiologists and 4 radiology residents) who work in hospitals in Vietnam were invited to participate in the study which was conducted at a BREAST workshop in Hanoi in 2019. The average age of doctors was 32 years old and each reader had an average of 2-year experience in interpreting mammograms. The majority of observers were female (76%) and more than 50% of radiologists read less than 20 mammograms per week and 72% of participants mentioned that they self-learned of mammogram interpretation skills through books and online materials (Table 3).

**Table 3:** Details of participants.

Average age	32
Average years of reading mammograms	2
**Working positions**	
Radiologists	21 (84%)
Radiology residents	4 (16%)
**Gender**	
Male	6 (24%)
Female	19 (76%)
**Case reading per week**	
< 20	14 (56%)
20–59	6 (24%)
60–100	5 (20%)
**Method of learning reading mammograms**	
From senior colleagues/attending training course	7 (28%)
Self-learning	18 (72%)

### Reading conditions

A reading room was set up and standardized to replicate a clinical setting with ambient lighting no greater than 20 lux which was measured via a photometer. Dual display workstations with a maximum display luminance of 600 cd/m^2^ and a spatial resolution of 2048 × 2560 pixels were used to show mammograms in DICOM format. The monitors were calibrated to the Grayscale Standard Display (DISCOM GSDF) to ensure workstations demonstrating the same image display quality. The workstations were embedded with display protocols similar as in clinical practice to provide readers with various levels of spatial resolution. The mammograms were displayed in 1 × 4 mode with the order of views being RMLO, LMLO, RCC, LCC from the left to the right. Additionally, each participant was provided with access to various post-processing tools including zooming, windowing/levelling and panning mechanisms to optimise image quality. The keypad was available for readers to switch to full-screen display of each mammogram view. Participants could also use the electronic magnifying glass to zoom in a section of a mammogram and alter the contrast and brightness of breast images.

### Reading procedure

Each participant was asked to read two sets of mammograms. The first one was the special training set and the second was the original BREAST set as described above. In each set, the reader was prompted to view the mammograms and localise detected abnormal lesions on each mammogram as well as give each marked lesion a score from two to five indicating the case from benign to absolute malignant where: 2 = benign; 3 = equivocal; 4 = suspicious of malignancy; 5 = highly suggestive of malignant. When there was no significant abnormality, the mammogram was recorded as a score of 1. Data collection was not undertaken under any time constraints and readers were allowed to go back to previous cases and edit marked lesions. Each set took participants no more than 3 h to complete. The BREAST software was used to record interactions of radiologists with mammograms and the time they spent on reading these images. Participants were trained to use the software prior to reading sessions and technical help was provided in a timely manner in person. Demographic and clinical experience information was obtained from each participant via an electronic questionnaire displayed on the screen prior to the commencement of the first reading session.

When a participant completed the first set, the system displayed immediate feedback to him or her which included the marking of the participant on mammograms compared with the truth of cancer locations and cancer types which helped the reader identify individual-based errors. The participant was given the time to go through the answers of each case and was asked to come back for reading the second set a day after to undertake a similar reading procedure as the first set. The observers were not informed about the number of cancers as well as nature of the lesions in each set until the test set was completed although they were aware that these mammogram sets were enriched with cancer cases.

### Statistical analysis

Case sensitivity, lesion sensitivity, specificity, receiver operating characteristics (ROC) area under the curve (AUC) and Jackknife alternative free response receiver operating characteristics (JAFROC) Figure of Merits (FOM) were calculated for each participant in each reading session. Case sensitivity was defined as the proportion of abnormal cases correctly identified by a reader when he or she marked a lesion with a recall rating (score 3, 4 or 5) and lesion sensitivity was the rate of cancer locations (regions of interest) correctly identified with the recall ratings. Specificity was calculated as the number of cancer-free mammograms correctly found by the radiologist with a non-recall rating (score 1 or 2) over the total number of normal cases. ROC was obtained by analysing case sensitivity and specificity whilst JAFROC was calculated through lesion sensitivity, specificity and ratings^24^. A lesion was considered as localized correctly when the center point of the lesion marked was within the radius of a true cancer location. Readers could mark multiple lesions on the mammograms but only the highest malignancy rating was used for data analysis.

In addition to the above metrics, the time participants spent on each set, the recall rate and the detection rate for stellate or spiculated masses were also analysed. The performance metrics of each participant in the first and second reading sessions were compared using the Wilcoxon Signed Ranks Test. The cancer detection rates of radiologists with different clinical experiences and characteristics were also analysed. The inter-expert reliability of radiologists in performance metrics was explored separately for each set via intra-class correlation coefficient test. The statistical tests were performed through the SPSS version 25.0 software (Chicago, IL, USA).
